# Supplementary material for: Detection of Aldehydes in Meat Products Based on Sulfonated Polystyrene Microspheres Modified with 2,4-Dinitrophenylhydrazine as Membrane-Protected Solid-Phase-Extraction Adsorbents
Source: Foods. 2025 Dec 29;15(1):101. doi: 10.3390/foods15010101 (PMC12786206; doi:10.3390/foods15010101)
Supplement: Supplementary file 1 [file foods-15-00101-s001.zip › foods-4027236-supplementary.pdf]

Supplementary material

Detection of Aldehydes in Meat Products Based on Sulfonated Polystyrene Microspheres Modified with 2,4-Dinitrophenylhydrazine as Membrane-Protected Solid-Phase-Extraction Adsorbents

Siyi Wang [1], Shibing Zhang [1], Min Fu [1], Siying Lu [1] and Qi Zhao [1,2,*]

[1] State Key Laboratory of Marine Food Processing and Safety Control, Dalian Polytechnic University, Dalian 116034, China; www011478@163.com (S.W.); 13470306550@163.com (S.Z.); 15905415700@163.com (M.F.); 15895679280@163.com (S.L.)

[2] National Engineering Research Center of Seafood, School of Food Science and Technology, Dalian Polytechnic University, Dalian 116034, China

* Correspondence: zhaoqi@dlpu.edu.cn; Tel.: +86-411-86323453

Table S1. The response values for evaluation of three variables based on Box-Behnken design.

Experiments	Factors			Recoveries (%)									
	Addition amount	Surface area	Quantity	Crotonaldehyde	Butanal	Pentanal	Trans-2-Hexanal	Trans, trans-2,4-Heptadienal	Hexanal	Heptanal	Octanal	Trans,trans-2,4-Decadienal	Nonanal
1	10.00	4	2	71	69	75	83	69	100	84	76	69	62
2	10.00	2.5	1	74	74	79	84	80	97	88	80	74	84
3	50.00	2.5	2	69	63	75	92	73	102	75	73	19	54
4	50.00	2.5	2	69	63	75	92	73	102	75	73	19	54
5	10.00	2.5	3	73	68	73	90	85	101	92	92	75	93
6	50.00	2.5	2	69	63	75	92	73	102	75	73	19	54
7	90.00	1	2	69	46	60	93	65	82	71	55	71	43
8	50.00	1	1	71	61	69	95	73	89	82	77	25	79
9	50.00	1	3	67	59	68	90	62	91	75	74	19	57
10	50.00	4	3	79	66	76	94	98	108	95	92	53	81
11	50.00	4	1	87	82	86	102	94	114	97	90	53	86
12	50.00	2.5	2	69	63	75	92	73	102	75	73	19	54
13	10.00	1	2	69	65	72	82	75	86	82	76	68	77
14	90.00	2.5	1	77	48	61	98	94	85	85	90	99	88
15	90.00	4	2	73	58	61	84	26	79	46	21	27	48
16	5.00	2.5	2	69	63	75	92	73	102	75	73	19	54
17	90.00	2.5	3	52	48	62	83	69	75	72	58	31	59

Table S2. The intra- and inter-day precisions and recoveries of aldehydes in the spiked chicken samples

Aldehydes	intra-day precision						inter-day precision					
	5 ng g^{-1}		50 ng g^{-1}		200 ng g^{-1}		5 ng g^{-1}		50 ng g^{-1}		200 ng g^{-1}	
	recoveries (%)	RSD (%)	recoveries (%)	RSD (%)	recoveries (%)	RSD (%)	recoveries (%)	RSD (%)	recoveries (%)	RSD (%)	recoveries (%)	RSD (%)
Crotonaldehyde	86	3	94	7	90	1	84	9	89	7	90	3
Butanal	68	4	85	6	74	1	67	2	82	5	74	3
Pentanal	74	4	86	7	86	2	76	11	85	9	87	3
Trans-2-Hexanal	84	4	96	3	96	4	86	8	94	6	98	2
Trans,trans-2,4-Heptadienal	61	2	89	9	103	3	71	6	101	5	100	8
Hexanal	74	2	106	10	97	2	76	9	105	6	98	1
Heptanal	74.88	5	96	3	96	4	68	7	96	6	97	5
Octanal	72	4	86	6	89	3	70	3	91	8	84	6
Trans,trans-2,4-Decadienal	58	2	73	7	72	7	56	6	72	4	72	3
Nonanal	75	8	83	7	85	9	73	2	87	6	84	7

Table S3. Parameters of Box-Behnken design experiment

Aldehydes	β_0	β_1	β_2	β_3	β_{12}	β_{13}	β_{23}	β_{11}	β_{22}	β_{33}	F-value	P-value	R^2	Predicted recoveres（%）
Crotonaldehyde	68.75	-2.17	4.31	-4.70	0.47	-5.97	-1.02	-2.62	4.54	2.91	4.01	0.0404	0.8374	83.2262
Butanal	62.51	-9.45	5.61	-2.80	2.12	1.43	-3.31	-5.21	2.01	2.26	14.82	0.0009	0.9501	81.6979
Pentanal	74.58	-6.85	3.61	-2.24	-0.45	1.69	-2.10	-6.80	-0.55	0.79	9.11	0.0041	0.9213	85.6154
Trans-2-Hexanal	92.45	2.37	0.59	-2.63	-2.40	-5.26	-0.68	-6.70	-0.33	2.96	7.33	0.0078	0.9040	94.3223
Trans,trans-2,4-Heptadienal	73.33	-6.68	1.44	-3.42	-8.35	-7.35	3.62	-7.40	-7.21	15.85	1.18	0.4247	0.6020	85.2005
Hexanal	102.36	-7.87	6.56	-1.34	-4.49	-3.49	-2.10	-13.21	-2.20	0.29	9.48	0.0036	0.9242	111.443
Heptanal	74.94	-8.96	1.50	-2.31	-6.91	-4.19	1.28	-3.56	-0.66	13.11	2.68	0.1036	0.7751	95.1167
Octanal	72.65	-12.54	-0.34	-2.55	-8.62	-10.86	1.18	-9.24	-6.28	16.73	3.18	0.0707	0.8034	86.9858
Trans,trans-2,4-Decadienal	19.47	-7.30	2.27	-9.00	-11.27	-17.16	1.64	35.73	3.39	14.75	3.93	0.0424	0.8348	68.0404
Nonanal	54.40	-9.74	2.61	-5.84	5.14	-9.49	4.01	4.44	-1.13	22.44	11.86	0.0018	0.9385	78.8076

Table S4. Recoveries of 10 aldehydes in different spiked barbecue products (50ng g^{-1}).

Aldehydes	Pork		Beef		Mutton		Chicken		Chicken wings		Fish	
	Content (ng g^{-1})	Recovery (%)	Content (ng g^{-1})	Recovery (%)	Content (ng g^{-1})	Recovery (%)	Content (ng g^{-1})	Recovery (%)	Content (ng g^{-1})	Recovery (%)	Content (ng g^{-1})	Recovery (%)
Crotonaldehyde	43.49±0.21	87±0	47.75±3.30	96±7	45.64±0.18	91±0	47.80±2.12	96±4	46.61±0.26	93±1	45.82±5.70	92±11
Butanal	46.22±5.21	92±10	48.51±4.35	90±9	45.06±1.87	90±4	42.78±3.99	86±8	61.20±1.47	90±3	44.74±3.63	89±7
Pentanal	114.08±2.02	86±4	152.93±5.02	87±10	69.77±1.05	86±2	68.10±3.24	90±6	208.42±1.96	88±4	128.60±1.58	91±3
Trans-2-Hexanal	46.11±2.83	92±6	48.42±1.46	94±3	45.58±0.11	91±0	48.15±1.37	96±3	46.61±2.43	90±5	51.46±0.76	103±2
Trans,trans-2,4-Heptadienal	39.08±1.50	78±3	61.29±4.09	82±8	42.71±0.86	85±2	41.17±0.60	82±1	40.96±3.27	82±7	42.50±4.00	85±8
Hexanal	92.43±5.09	102±10	366.0±4.73	110±9	70.44±7.03	107±14	65.31±1.01	99±2	135.76±5.86	109±12	66.94±5.81	106±12
Heptanal	56.89±2.67	105±5	88.48±2.29	89±5	51.12±1.57	102±3	53.07±3.96	106±8	50.22±3.59	86±7	52.06±7.17	94±14
Octanal	54.21±2.24	88±4	91.89±5.46	86±11	51．29±2.51	87±5	43.31±3.62	87±7	61.05±2.66	89±5	50.74±3.80	93±8
Trans,trans-2,4-Decadienal	61.03±0.88	71±2	131.27±4.31	77±9	35.51±1.31	71±3	36.22±1.30	72±3	148.02±1.51	76±3	36.48±0.34	71±1
Nonanal	122.16±4.57	88±9	174.31±4.52	88±9	175.06±2.90	94±6	75.42±2.71	87±5	122.25±4.72	86±9	100.58±2.51	86±5
